# Quantitative Fluorescent *in situ* Hybridization Reveals Differential Transcription Profile Sharpening of Endocytic Proteins in Cochlear Hair Cells Upon Maturation

**DOI:** 10.3389/fncel.2021.643517

**Published:** 2021-02-26

**Authors:** Guobin Huang, Stephanie Eckrich

**Affiliations:** Center for Integrative Physiology and Molecular Medicine, School of Medicine, Department of Biophysics, Saarland University, Homburg, Germany

**Keywords:** fluorescent *in situ* hybridization, cochlea, hair cells, endocytosis, dynamin-1, dynamin-3, endophilin-A1, terminal differentiation

## Abstract

The organ of Corti (OC) comprises two types of sensory cells: outer hair cells (OHCs) and inner hair cells (IHCs). While both are mechanotransducers, OHCs serve as cochlear amplifiers, whereas IHCs transform sound into transmitter release. Reliable sound encoding is ensured by indefatigable exocytosis of synaptic vesicles associated with efficient replenishment of the vesicle pool. Vesicle reformation requires retrieval of vesicle membrane from the hair cell’s membrane via endocytosis. So far, the protein machinery for endocytosis in pre-mature and terminally differentiated hair cells has only partially been deciphered. Here, we studied three endocytic proteins, dynamin-1, dynamin-3, and endophilin-A1, by assessing their transcription profiles in pre-mature and mature mouse OCs. State-of-the-art RNAscope^®^ fluorescent *in situ* hybridization (*FISH*) of whole-mount OCs was used for quantification of target mRNAs on single-cell level. We found that pre-mature IHCs contained more mRNA transcripts of *dnm1* (encoding dynamin-1) and *sh3gl2* (endophilin-A1), but less of *dnm3* (dynamin-3) than OHCs. These differential transcription profiles between OHCs and IHCs were sharpened upon maturation. It is noteworthy that low but heterogeneous signal numbers were found between individual negative controls, which highlights the importance of corresponding analyses in RNAscope^®^ assays. Complementary immunolabeling revealed strong expression of dynamin-1 in the soma of mature IHCs, which was much weaker in pre-mature IHCs. By contrast, dynamin-3 was predominantly found in the soma and at the border of the cuticular plates of pre-mature and mature OHCs. In summary, using quantitative RNAscope^®^ FISH and immunohistochemistry on whole-mount tissue of both pre-mature and mature OCs, we disclosed the cellular upregulation of endocytic proteins at the level of transcription/translation during terminal differentiation of the OC. Dynamin-1 and endophilin-A1 likely contribute to the strengthening of the endocytic machinery in IHCs after the onset of hearing, whereas expression of dynamin-3 at the cuticular plate of pre-mature and mature OHCs suggests its possible involvement in activity-independent apical endocytosis.

## Introduction

The mammalian hearing organ, the organ of Corti (OC) contains two types of mechanosensory hair cells (HCs), inner HCs (IHCs) and outer HCs (OHCs), which possess hair-like stereocilia protruding from the actin-rich cuticular plate located at the apical border of the cell. Sound-induced deflection of the stereocilia causes depolarization of HCs.

Inner hair cells, the genuine receptor cells of the OC, generate graded receptor potentials and are capable of indefatigably converting acoustic signals with high temporal acuity into action potentials in myelinated type I afferent nerve fibers (NFs) of spiral ganglion neurons (SGNs) (Rutherford and Moser, 2016). This is achieved by the unique vesicle machinery at the specialized ribbon synapses located at the basolateral membrane of IHCs, which lack many proteins essential for transmitter release at central synapses (Beurg et al., 2010; Johnson et al., 2010; Nouvian et al., 2011; Vogl et al., 2015). Instead, IHCs express otoferlin, which is essential for hearing and involved in multiple processes at the release site, such as scaffolding of Ca_*v*_1.3 channels, Ca^2+^ sensing of exocytosis, tethering of vesicles and vesicle pool replenishment, and clathrin-mediated endocytosis (reviewed in Pangršič et al., 2015; Moser and Starr, 2016).

Outer hair cells are electromotile and mainly serve as cochlear amplifiers via sound-induced contraction of the cell body. They possess few, small ribbon synapses innervated by postsynaptic terminals of unmyelinated type II SGN fibers representing a minority of 5–10% in the total SGN population (Spoendlin, 1985). OHC synapses only respond to stimuli of high intensity (Weisz et al., 2012) and might serve as nociceptors detecting acoustic trauma in the OC (Flores et al., 2015; Liu et al., 2015; Wood et al., 2020).

Before the onset of hearing at postnatal day 12 (P12) in most rodents, HCs undergo a period of terminal differentiation characterized by their profound remodeling including up- and downregulation of various ion channels, refinement of IHC synaptic function, and acquisition of OHC electromotility. OHCs reach functional maturity at P7-8 (Marcotti and Kros, 1999; Abe et al., 2007) and IHCs at around the onset of hearing (Kros et al., 1998; Marcotti et al., 2003). During this period, both IHCs (Kros et al., 1998) and OHCs (Beurg et al., 2008; Ceriani et al., 2019) generate sensory-independent action potentials, which are required for refinement of the auditory pathway (Katz and Shatz, 1996; Blankenship and Feller, 2010).

The generation of action potentials in pre-mature IHCs and OHCs and particularly sustained neurotransmission in mature sound-transmitting IHCs require efficient reformation of the synaptic vesicle pool, which includes rapid activity-dependent retrieval of the fused vesicle from the presynaptic membrane (Wittig and Parsons, 2008). To date, endocytosis in pre-mature IHCs is poorly understood; however, lower expression rates of endocytic proteins (Roux et al., 2006; Duncker et al., 2013) suggest that endocytosis undergoes a period of terminal differentiation similar to the rest of the cell. The mechanisms and proteins assuring efficient endocytosis and vesicle pool replenishment upon maturation of IHCs are only partially deciphered: Dynamin-1, a GTPase mediating fission of vesicular membrane during endocytosis, promotes replenishment of the synaptic vesicle pool of IHCs (Duncker et al., 2013; Neef et al., 2014). Conflicting data found expression of dynamin-3 exclusively in stereocilia of OHCs (Liu et al., 2014; Li et al., 2018), whereas Neef et al. (2014) showed dynamin-3 in the soma of IHCs. Dynamins interact with the membrane-binding and curvature-inducing endophilins (Sundborger and Hinshaw, 2014), whose isoform endophilin-A1 is required for vesicle retrieval in IHCs (Kroll et al., 2019).

In contrast to IHCs, our knowledge of synaptic endocytosis at the basolateral membrane of OHCs is sparse, whereas activity-independent endocytosis at their cuticular plates is possibly involved in cell maintenance rather than synaptic release (Griesinger et al., 2004; Harasztosi and Gummer, 2019).

We here studied terminal differentiation of the endocytic machinery of IHCs that ensure highly efficient endocytosis of IHCs in the mature cochlea. To this end, we analyzed mRNA transcription profiles of three endocytic genes *dnm1*, *dnm3*, and *sh3gl2* encoding dynamin-1, dynamin-3, and endophilin-A1, respectively, in murine pre-mature and mature whole-mount OCs using state-of-the-art RNAscope^®^ fluorescent *in situ* hybridization (*FISH*), which revealed subcellular localization of single mRNA molecules. The well-defined shape of both IHCs and OHCs, which are small, round cells without any nerve processes, allowed quantification of the entire mRNA content of individual cells. Immunohistochemistry was employed to uncover the associated protein expression and localization of dynamin-1 and -3 in the OC.

## Materials and Methods

### Animals

Pre-hearing mice were used at postnatal day 4 and compared to 3-week-old animals at postnatal day 20, i.e., about 1 week after the onset of hearing. Young adult mice were anesthetized using isoflurane. For both age groups, inner ears were dissected after mice of either sex had been sacrificed by cervical dislocation. Animals were housed at an ambient temperature of 22°C and a 12 h light/dark cycle with free access to food and water. All procedures were in accordance with the European Communities Council Directive (86/609/EEC) and approved by the regional board for scientific animal experiments of the Saarland, Germany.

### RNAscope^®^ Fluorescent *in situ* Hybridization

#### Day 1—Tissue Pre-treatment

Inner ears were removed from the temporal bone and immediately immersed in 4% paraformaldehyde (PFA) in 1× phosphate-buffered saline (PBS). Rapid penetration of the tissue with fixative was increased by carefully injecting 1 ml 4% PFA into the round and oval window of mature cochleae following removal of a small bone chip at the helicotrema to ease the exit of solution. Moreover, after 10 min of immersion in fixative, the cochlear bone or cartilage was partly removed for both 3-week-old and 4-day-old mice, respectively. Pre-mature inner ears were fixed for 1 h and mature tissue for 2 h at room temperature (RT) on a shaker. Cochleae were then washed for 3 × 10 min in 0.1% Tween-20 in 1× PBS (PBT), subsequently dehydrated at RT in a graded methanol (MeOH) series (50, 75, and 100% in PBT, 10 min for each grade), and stored for up to 2 weeks in 100% MeOH at −20°C.

#### Day 2—RNAscope^®^ Fluorescent *in situ* Hybridization

All solutions and probes for the RNAscope^®^ assay were acquired from Advanced Cell Diagnostics (United States) and prepared according to the instructions of the manufacturer. Before each use, probes were pre-warmed to 40°C for 10 min and then cooled down to RT; Protease III solution and Amplifiers I-IV were allowed to equilibrate to RT and mixed by inverting the tube. We used target probes for *dnm1* (446931; detection channel-1), *dnm3* (451841-C2; detection channel-2) and *sh3gl2* (492641-C3; detection channel-3); positive control probes (320881) for *polr2a* (detection channel-1), *ppib* (detection channel-2) and *ubc* (detection channel-3); as well as negative control probes for bacterial *dapB* (320871; detection channels 1–3). Unless otherwise stated, tissue was washed with RNAscope^®^ washing buffer and amplification was carried out using Amp4 “Alt-B” solution with the following combinations of detection channel and fluorophore: channel-1 + Atto 550; channel-2 + Alexa 488; and channel-3 + Alexa 647. Protease digestion, washing, and DAPI staining were performed at RT on a shaker. Probe hybridization and signal amplification were carried out at 40°C in an incubator.

Before hybridization, cochleae were rehydrated at RT in a reverse MeOH series (100, 75, and 50% in PBT; 10 min for each grade) and washed 5 × 6 min in PBT. During washing, the apical-turn OC was dissected in PBT and the tectorial membrane was removed. Tissue was placed in a basket modified from a mini cell strainer (pluriStrainer Mini 40 μm, 43-10040, pluriSelect Life Science, Germany), which was transferred between the wells of a 48-well plate. This was a very gentle approach for the delicate OC tissue; however, the small volume of the wells required more frequent exchange of the washing buffer in order to achieve sufficient rinsing and reduced background staining.

Organs of Corti were digested for 25 min (pre-mature tissue) and 12 min (mature) at RT in Protease III solution for cultured cells instead of Protease IV (for frozen tissue), as in our hands the latter digested most HCs and surrounding supporting cells even after brief incubation. Following washing for 5 × 3 min in PBT, 48-well plates were sealed using adhesive film to avoid evaporation and OCs were hybridized with the probe mixture for 2 h at 40°C. Note that hybridization times exceeding 8 h caused a high number of false positive dots in the negative controls. Subsequently, tissue was washed for 8 × 5.5 min with washing buffer, re-fixed for 10 min with 4% PFA, and washed again for 5 × 3 min. Probe signals were amplified by incubation at 40°C in Amp1 (35 min), Amp2 (20 min), Amp3 (35 min), and Amp4 solution (20 min); between each step, tissue was washed for 8 × 5.5 min in washing buffer. Following a final washing step for 5 × 3 min, nuclei were labeled with DAPI (1:333 in 1 × PBS) for 5 min (pre-mature) or 3 min (mature) and washed 3 × 1 min with 1 × PBS.

### Tissue Embedding

Different embedding media were tested, while we established RNAscope^®^ *FISH* with positive and negative control probes. As fluid media, we used VECTASHIELD^®^ Antifade Mounting Medium (H-1000, Vector Laboratories, United States), and ROTI^®^Mount FluorCare (HP19.1, Carl Roth GmbH + Co. KG, Germany). We also used the following solidifying media: FluorSave^TM^ Reagent (345789, Millipore, United States), Dako Fluorescence Mounting Medium (S3023, Dako, Denmark), and ProLong^TM^ Glass Antifade Mountant (P36982, Invitrogen, United States). For all target probes, tissue was embedded in FluorSave Reagent and allowed to cure for ≥ 1 h before analysis.

### Data Quantification

For each age group, *RNAscope* experiments were repeated in ≥ three animals. *FISH* signals were visualized and stored as a 16-bit image using a confocal laser-scanning microscope (LSM710, Zeiss, Germany). For each animal, ≥ five z-stack images were acquired encompassing the entire cell bodies of stretches of 10 IHCs and adjacent three rows of OHCs using a 63 × oil objective (Plan-Apochromat 63 × /1.4 Oil DIC, Zeiss, Germany). Pixel size was 0.07 μm, and the interval between slices was 0.32 μm. Note that the *FISH* puncta were too small to be reliably and individually detected at a lower magnification.

Fluorescently labeled mRNA was quantified using Fiji (Schindelin et al., 2012). Maximum intensity z-axis projections (MIZP) of 10 IHCs or, alternatively, 10 × 3 rows of OHCs were generated, which were then split into their individual detection channels for further analysis. Rolling ball background subtraction was performed with a rolling ball radius between 2 and 9 pixels. Thresholds were set between 8 and 17% of the full range. Following water shedding, particle analysis was performed with a minimum size of 0.02–0.03 μm^2^ and a maximum size of 0.3 μm^2^. For each image, the average number of mRNA molecules per hair cell was then determined by dividing the number of particles by the number of respective hair cells, i.e., 10 for IHCs or 30 for OHCs. For the target mRNAs but not for the positive controls, data were normalized to the median number of signals in the negative control of the respective experimental day and detection channel.

### Immunohistochemistry

Inner ears were removed from the temporal bone and immediately immersed in ethanol (EtOH). In pre-mature cochleae, the cochlear bone or cartilage was partly removed. In mature cochleae, rapid penetration of the tissue with fixative was increased by carefully injecting 1 ml EtOH into the round and oval window of cochleae following removal of a small bone chip at the helicotrema to ease the exit of solution. Tissues at both ages were fixed for 20 min at −20°C. OC was dissected, placed on an objective slice and washed for 10 min with 1 × PBS, permeabilized for 10 min with 0.5% Triton X-100 in 1 × PBS, and blocked for 60 min with 1% bovine serum albumin (BSA) in 1 × PBS at RT. Subsequently, OCs were incubated at 4°C overnight with primary antibodies directed against dynamin-1 (monoclonal mouse, 1:200, 610245, BD, United States), ap-180 (polyclonal rabbit, 1:500, 155003, Synaptic Systems GmbH, Germany), dynamin-3 (polyclonal rabbit, 1:2000, 115302, Synaptic Systems GmbH, Germany), and myosin-7a (monoclonal mouse, 1:50, sc-74516, Santa Cruz Biotechnology, United States) in reaction buffer containing 0.5% BSA and 0.2% Triton X-100 in 1 × PBS. After being washed for 2 × 15 min with 0.1% Triton X-100 in 1 × PBS, tissue was incubated at RT for 70 min with secondary antibodies (Alexa 568-goat α-mouse, 1:500, A-11019, Invitrogen, United States; Alexa 488-donkey α-rabbit, 1:500, ab150073, Abcam, United Kingdom) in reaction buffer and washed 2 × 15 min with 0.1% Triton X-100 in 1 × PBS at RT. Following incubation with 1 × PBS for 5 min, nuclei were labeled with DAPI (1:333 in 1 × PBS) for 5 min and washed for 3 × 1 min in 1 × PBS. Images were obtained as described for RNAscope^®^ *FISH* using a 20 × objective (Plan-Apochromat 20 × /0.8, Zeiss, Germany) and a 63 × oil objective (Plan-Apochromat 63 × /1.4 Oil DIC, Zeiss, Germany). Images were processed using Fiji, and the reslice function was used to create maximum intensity *X*-axis projections (MIXP). All immunohistochemical experiments were repeated ≥ three times.

### Statistics

Statistical analysis was performed using SPSS (IBM Corp., United States). Quantitative data are given as median ± SD and presented as boxplot with median, 25–75% confidence interval (box); 5–95% confidence interval (whiskers); and mean (×). Outliers shown in boxplots exceeded the tolerance of 1.5 × interquartile range. We used Mann–Whitney *U* test for comparison of two age groups and Friedman test followed by Dunn–Bonferroni *post hoc* analysis for larger group numbers, because not all groups were normally distributed, with a significance level of *p* < 0.05.

## Results

### RNAscope^®^ Fluorescence *in situ* Hybridization in the Whole-Mount Organ of Corti

RNAscope^®^ *FISH* allows the simultaneous use of three or more mRNA probes, which are assigned to detection channels and labeled by channel-specific fluorophores. According to the manufacturer, each *FISH* dot represents a single mRNA molecule allowing cell-specific mRNA quantification. In order to quantitatively analyze developmental changes in transcription profiles of endocytic proteins, we aimed at establishing RNAscope^®^ *FISH* on OCs of 4-day- and 3-week-old animals with the best possible signal-to-noise ratio.

The assay was established in our lab using positive control probes suggested by the manufacturer, which are specific for mRNA of housekeeping genes typically expressed at low, moderate, and moderate-high levels: *polr2a* (encoding RNA polymerase II subunit A, low expression) in detection channel-1, *ppib* (peptidylprolyl isomerase B, moderate expression) in detection channel-2, and *ubc* (ubiquitin C, moderate-high expression) in detection channel-3, which were visualized with Atto 550, Alexa 488, and Alexa 647 fluorophores, respectively. Additionally, OCs hybridized with probes specific for bacterial *dapb* (dihydrodipicolinate reductase) mRNA served as negative control for all three detection channels. Due to profound differences in the morphology and texture of the pre-mature compared to the mature cochlear tissue, we adjusted times of fixation and protease digestion for each age group in a way that resulted in the highest possible signal number in the positive controls, while concomitantly keeping the number of non-specific puncta in the negative controls as low as possible (see “Materials and Methods” section “RNAscope^®^ Fluorescent *in situ* Hybridization”). Conditions of the *FISH*, including duration of hybridization, were otherwise identical. Preliminary experiments showed that the choice of the mounting medium affected the quality of the fluorescent signal and the longevity of the tissue (Supplementary Figure 1), leading us to use the same mounting medium, FluorSave, for all experiments.

**FIGURE 1:**
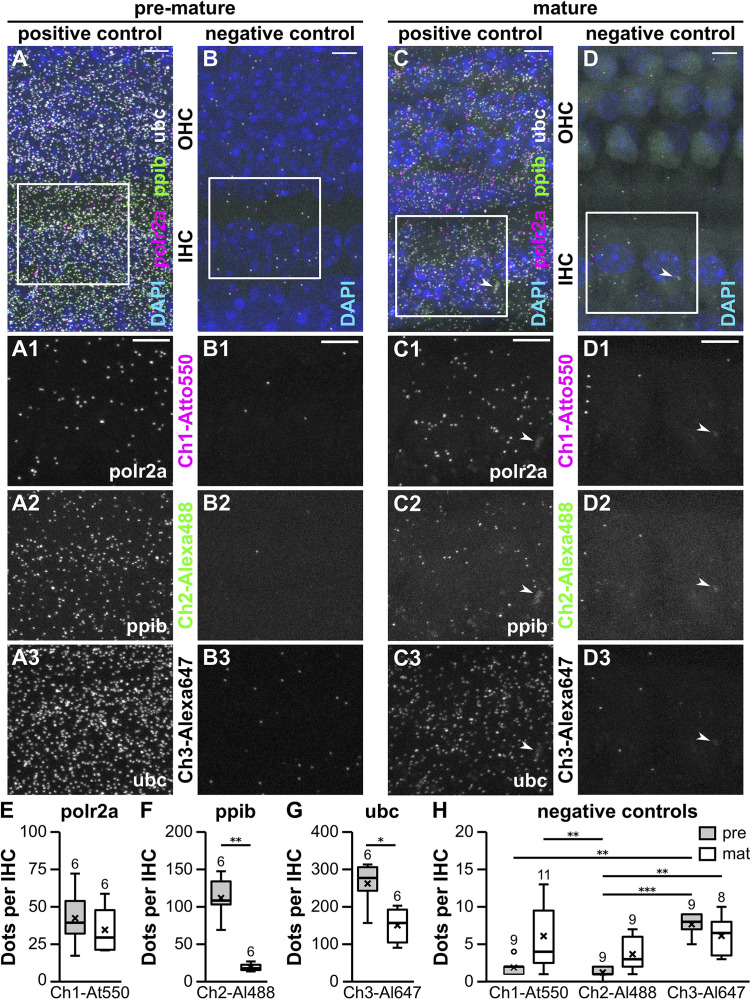
Whole-mount RNAscope^®^ fluorescent *in situ* hybridization of pre-mature and mature apical-turn organs of Corti. MIZP of a typical pre-mature (P4; **A,B**) and a mature whole-mount OC (P20; **C,D**). Positive control probes specific for the endogenous housekeeping genes *polr2a*, *ppib*, and *ubc* were labeled with Atto 550, Alexa 488, and Atto 647, respectively **(A,C)**. Probes targeting the bacterial gene *dapB* served as negative controls for all three detection channels (Ch1–Ch3, **B,D**). Merged overview images show three IHCs and three adjacent rows of OHCs with DAPI-labeled nuclei (**A–D**, top). *FISH* signals are shown for two IHCs (frame in **A**–**D**) at a higher magnification for the individual positive controls *polr2a* **(A1,C1)**, *ppib* **(A2,C2)**, and *ubc* **(A3,C3)**, as well as their respective negative controls **(B1–3,D1–3)**. Arrowheads: non-specific blurry staining present in all detection channels was exclusively found at mature IHCs. *FISH* signals were quantified in stretches of IHCs and normalized to counts per IHC before (pre-mature, gray) and after the onset of hearing (mature, white, **E–H**). Positive control probes detected expression of all housekeeping genes in pre-mature IHCs with *polr2a* < *ppib* < *ubc* (note the axis labeling). Comparison of both age groups by Mann–Whitney *U* test revealed an age-dependent reduction of *ppib* and *ubc*, but not *polr2a* abundance **(E–G)**. Signal numbers of the negative control probes were generally low. For individual detection channels, signal numbers were similar between age groups, but partly differed between detection channels in pre-mature IHCs **(H)**. Numbers above each boxplot represent the number of images analyzed. *p < 0.05, **p < 0.01, ***p < 0.001. Scale bars: 5 μm.

Figure 1 shows MIZP of confocal images taken at 63× magnification from three IHCs and the adjacent three rows of OHCs in the pre-mature OC of a 4-day-old (Figures 1A,B) and the mature OC of a 3-week-old mouse (Figures 1C,D). In both age groups, puncta of all three positive control probes were distributed across the tissue with no prevalence to a certain cell type (Figures 1A,C), which was also evident from a MIXP (Supplementary Figure 2). In contrast, only few signals were present in the negative control probes (Figures 1B,D). We sporadically observed blurry fluorescent patches located in the IHC region of mature OCs (Figures 1C,D, arrowheads), which were simultaneously present in all detection channels of both, positive and negative controls, and therefore considered non-specific. A detailed view of IHCs shows that, for both age groups, the number of *FISH* dots differed between the individual channels of the positive (Figures 1A1–3,C1–3) as well as between the negative controls (Figures 1B1–3,D1–3).

RNAscope^®^ dots were quantified in MIZP images containing stretches of 10 IHCs using particle analysis in Fiji (see “Materials and Methods” section “Data Quantification”) and then normalized to one IHC (Figures 1E–H). The median number of puncta per pre-mature IHC increased from *polr2a* with 39.5 ± 18.9, to *ppib* with 108.5 ± 27.3 and *ubc* with 277.0 ± 57.4 puncta (*n* = 6 images). When comparing to mature IHCs (*n* = 6 images), we found that *polr2a* signals were similar between pre-mature and mature cells (29.5 ± 15.7; *p* = 0.59, Mann–Whitney *U* test; Figure 1E), whereas a developmental reduction was found for *ppib* (18.5 ± 4.8; *p* = 0.002, Mann–Whitney *U* test; Figure 1F) and *ubc* (157.0 ± 47.8; *p* = 0.015, Mann–Whitney *U* test; Figure 1G). For negative controls (Figure 1H), the number of fluorescent dots per IHC was generally low but significantly differed between detection channels and age groups [Friedman’s test: χ^2^(5) = 29.16, *p* < 0.001]. Comparison of the individual groups by Dunn–Bonferroni pairwise comparison (Table 1) revealed that for each of the detection channel (channels 1–3), the signal numbers of the negative control were similar between pre-mature and mature IHCs. Moreover, the negative-control signals in mature IHCs did not differ between detection channels, whereas in pre-mature IHCs, a significant difference was found between channel-1 and -3 (*p* = 0.032) and between channel-2 and -3 (*p* < 0.001).

**TABLE 1:** Dunn–Bonferroni test of pairwise comparison between negative controls for detections channel-1 (ch-1), channel-2 (ch-2), and channel-3 (ch-3) of pre-mature and mature IHCs following related-samples Friedman’s two-way analysis of variance with χ^2^(5) = 29.16, *p* < 0.001. Significant differences with *p* < 0.05 indicated by bold fonts and asterisks.

		Pre-mature		Mature		
		Ch-2 Alexa 488	Ch-3 Alexa 647	Ch-1 Atto 550	Ch-2 Alexa 488	Ch-3 Alexa 647
Pre-mature	Ch-1 Atto 550	*p* = 1.00	*p* = 0.032*	*p* = 0.167	*p* = 1.00	*p* = 0.167
	Ch-2 Alexa 488		*p* < 0.001*	*p* = 0.002*	*p* = 0.242	*p* = 0.002*
	Ch-3 Alexa 647			*p* = 1.00	*p* = 0.790	*p* = 1.00
Mature	Ch-1 Atto 550				*p* = 1.00	*p* = 1.00
	Ch-2 Alexa 488					*p* = 1.00
	Ch-3 Alexa 647					

*The respective fluorophore is given for each channel.*

In summary, robust *FISH* signals in the positive control revealed differential transcription rates of the housekeeping genes *polr2a*, *ppib*, and *ubc* between ages and probes. The number of fluorescent puncta was generally low in the negative control, but in one case reached 15% of the positive control. This might result in an overestimate of the actual mRNA numbers especially when analyzing genes with low transcript numbers. Hereafter, we thus normalized the number of signals resulting from our target probes to the negative controls by subtracting the median of the associated negative control. For individual detection channels, the signals of the negative controls never differed between pre-mature and mature IHCs, whereas differences were found between detection channels in pre-mature IHCs. In the following transcription analysis of endocytic mRNA, we thus decided not to compare the target mRNAs *dnm1*, *dnm3*, and *sh3gl2* to each other but strictly kept comparisons within the individual target mRNA.

### Age-Dependent Modification of *dnm1*, *dnm3*, and *sh3gl2* mRNA Transcription Profiles in Inner and Outer Hair Cells

**FIGURE 2:**
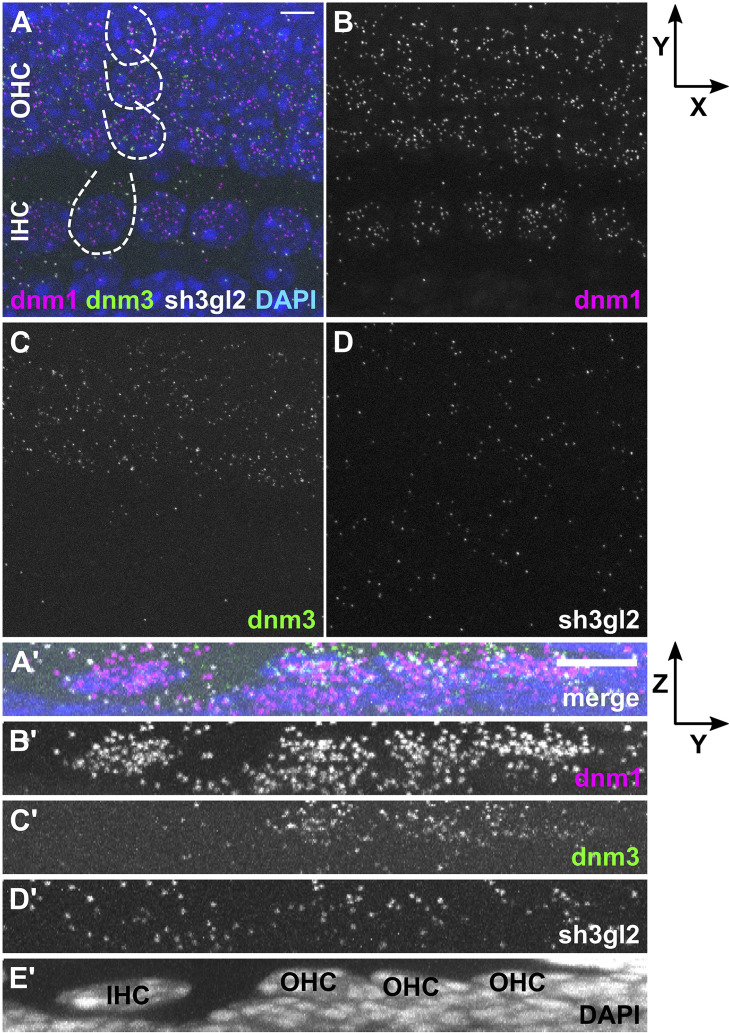
Differential expression of *dnm1*, *dnm3*, and *sh3gl2* mRNA in pre-mature inner and outer hair cells of neonatal mice. *FISH* using probes specific for mRNA of *dnm1*, *dnm3*, *and sh3gl2* was performed on the apical-turn whole-mount OC shown here for a typical neonatal mouse at P4. The merged MIZP of five IHCs and adjacent OHCs (partly indicated by dashed lines) shows that the *FISH* signal was mainly confined to HCs **(A)**. *Dnm1* and the less abundant *sh3gl2* mRNA were present in both IHCs and OHCs **(B,D)**, while *dnm3* was predominantly detected in OHCs **(C)**. **(A’–E’)** Maximum intensity X-projection of three IHCs and neighboring OHCs; note that the extension of the *Z*-axis is not true to scale. Scale bars: 5 μm.

**FIGURE 3:**
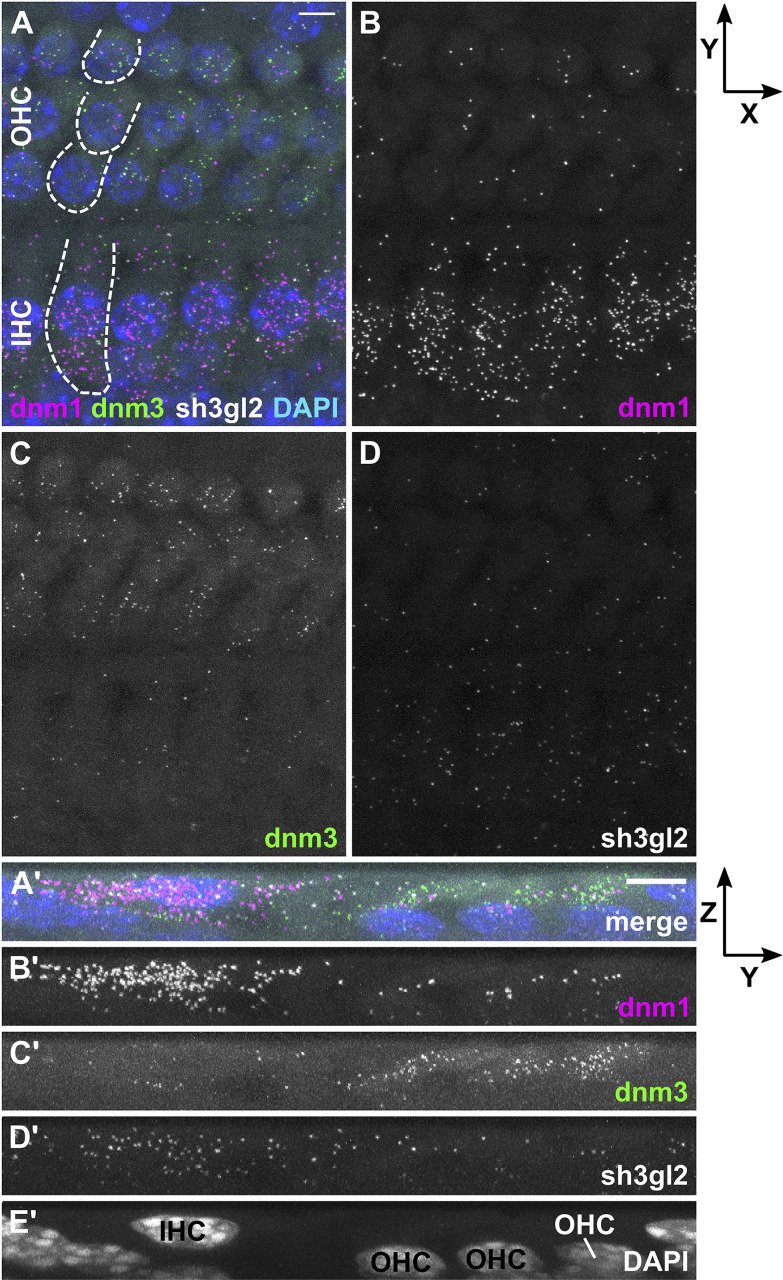
In the mature organ of Corti, *dnm1* and *sh3gl2* mRNA is predominantly found in IHCs, while *dnm3* is mostly present in OHCs. In a typical apical-turn OC of a hearing mouse at P20, *dnm1*, *dnm3*, and *sh3gl2* mRNA was mostly confined to the sensory cells **(A)**. *Dnm1* signals were mainly localized in IHCs, but also present in OHCs **(B)**, while *dnm3* dots were mostly found in OHCs **(C)**. *Sh3gl2* mRNA was detected in both HC types with a slightly higher amount in IHCs **(D)**. **(A’–E’)** Maximum intensity X-projection of three IHCs and neighboring OHCs; note that *Z*-axis is not true to scale. Scale bars: 5 μm.

In order to analyze transcription rates of endocytic genes in HCs before and after the onset of hearing, we employed RNAscope^®^ probes targeting mRNA of three endocytic proteins present in the mature cochlea: *dnm1* and *dnm3* encoding dynamin-1 and -3 (Neef et al., 2014; Li et al., 2018), as well as *sh3gl2* encoding endophilin-A1 (Kroll et al., 2019). mRNA signals of all three target probes were enriched in and restricted to IHCs and OHCs of both pre-mature and mature OCs, which was evident from *Z*- (Figures 2/3A–D) and *X*-axis collapsed projections (Figures 2/3A’–E’) and contrasts the even distribution of the positive control throughout the tissue (MIZP: Figure 1 and MIXP: Supplementary Figure 2). The enrichment of target mRNA signals in HCs but the absence of mRNA signals in the supporting cells, where activity-dependent endocytosis is not required, indicates a high specificity of the mRNA probes and allowed the quantification of mRNA in individual HCs. Analysis of the separate probes showed that *dnm1* (Figures 2B,B’) and—to a lesser extent—*sh3gl2* puncta (Figures 2D,D’) were present in both types of HCs, while *dnm3* was largely restricted to OHCs (Figures 2C,C’). After the onset of hearing, *dnm1* (Figures 3A,A’) and *sh3gl2* (Figures 3D,D’) showed a higher preference to IHCs than OHCs, while *dnm3* still was predominantly found in OHCs (Figures 3C,C’). All three mRNAs were additionally present in SGN somata, with a higher abundance of *dnm1* and *sh3gl2* as compared to the lower number of *dnm3* puncta (Supplementary Figure 3).

**FIGURE 4:**
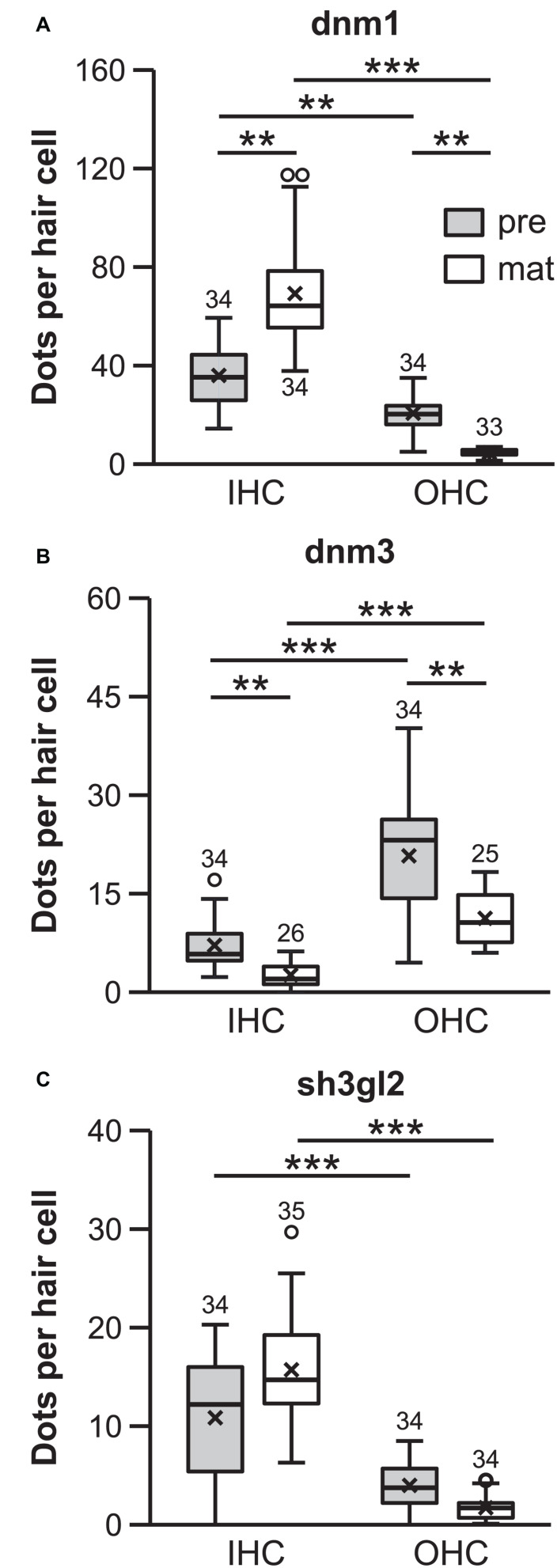
Quantification of *dnm1*, *dnm3*, and *sh3gl2 FISH* signals revealed differential transcription profiles of inner and outer hair cells. mRNA content differed between HC types and age groups for all genes analyzed (*dnm1*, *dnm3*, and *sh3gl2*: *p* < 0.001; Friedman test followed by Dunn–Bonferroni test for multiple comparisons; **A–C**). **p < 0.01, ***p < 0.001. All target probe signal numbers were normalized with respect to the number of fluorescent signals in their respective negative controls. Numbers above each boxplot represent the number of images analyzed.

In order to quantify the mRNA transcripts during terminal differentiation and after the onset of hearing, fluorescent signals of each target probe were analyzed separately. The number of dots was determined in stretches of 10 IHCs and, separately, in their adjacent 3 rows × 10 OHCs and the resulting transcript numbers were averaged to transcripts per one HC, followed by normalization to the signals of the respective detection channel of the matching negative control. For all three target mRNAs, the number of *FISH* puncta significantly differed between ages and cell types [Figure 4; *Dnm1*, χ^2^(3) = 99.0, *p* < 0.001; *dnm3*, χ^2^(3) = 56.3, *p* < 0.001; *sh3gl2*, χ^2^(3) = 68.2, *p* < 0.001; Friedman test].

For each target mRNA, individual groups were compared via Bonferroni-adjusted Dunn’s test of multiple comparisons. In the pre-mature OC, more *dnm1* mRNA was present in IHCs (35.3 ± 12.4, *n* = 34) than OHCs (20.3 ± 6.7, *n* = 34; *p* = 0.010; Figure 4A). Upon maturation, *dnm1* mRNA abundance was significantly increased to 64.3 ± 21.2 mRNA dots per IHC (*n* = 34; *p* = 0.010), whereas it was concomitantly reduced to 5.0 ± 1.4 dots in OHCs (*n* = 33; *p* = 0.010; Figure 4A) resulting in a 10-fold higher content of *dnm1* mRNA in mature IHCs that in their neighboring OHCs (*p* < 0.001).

In contrast, *dnm3* mRNA was predominately expressed in OHCs of both age groups, exceeding the *dnm3* content of IHCs by four to five times: pre-mature OHCs contained 23.2 ± 8.6 *dnm3* puncta (*n* = 34), whereas significantly less mRNA was found in IHCs at this age (5.8 ± 3.9; *n* = 34; *p* < 0.001; Figure 4B). In the mature tissue, while *dnm3* content was still significantly higher in OHCs than IHCs (*p* < 0.001), an age-dependent reduction of mRNA load was found for both OHCs (10.6 ± 3.8, *n* = 25; *p* = 0.015) and IHCs (2.0 ± 1.8, *n* = 25; *p* = 0.015; Figure 4B).

Similar to *dnm1*, expression levels of *sh3gl2* mRNA were higher in IHCs than OHCs at both ages analyzed. Pre-mature IHCs contained significantly more mRNA dots (12.2 ± 6.7; *n* = 34) than OHCs (3.8 ± 2.4, *n* = 34; *p* < 0.001, Figure 4C). For both cell types, the amount of mRNA was not significantly altered upon maturation (IHCs, 14.7 ± 4.9, *n* = 35; *p* = 0.325; OHCs, 1.7 ± 1.1, *n* = 34; *p* = 1.00, Figure 4C) and was still significantly higher in IHCs than OHCs (*p* < 0.001).

In summary, *dnm1* and *sh3gl2* mRNA content was generally higher in IHCs than OHCs. For *dnm1* mRNA, this transcription profile was sharpened upon maturation. In contrast, *dnm3* mRNA showed highest enrichment in pre-mature OHCs but was downregulated in both hair cell types upon maturation.

### Protein Expression and Localization of Dynamin-1 and -3 in the Pre-mature and Mature Organ of Corti

In order to corroborate our findings of mRNA transcription rates, we analyzed the expression and localization of the translated protein. OCs from the apical cochlear turn of 4-day- and 3-week-old mice were immunolabeled for dynamin-1 and -3 encoded by *dnm1* and *dnm3*, respectively. The protein expression of endophilin-A1 encoded by *sh3gl2* was not analyzed, because so far, no specific labeling could be established in the OC (own data, not shown; Kroll et al., 2019).

**FIGURE 5:**
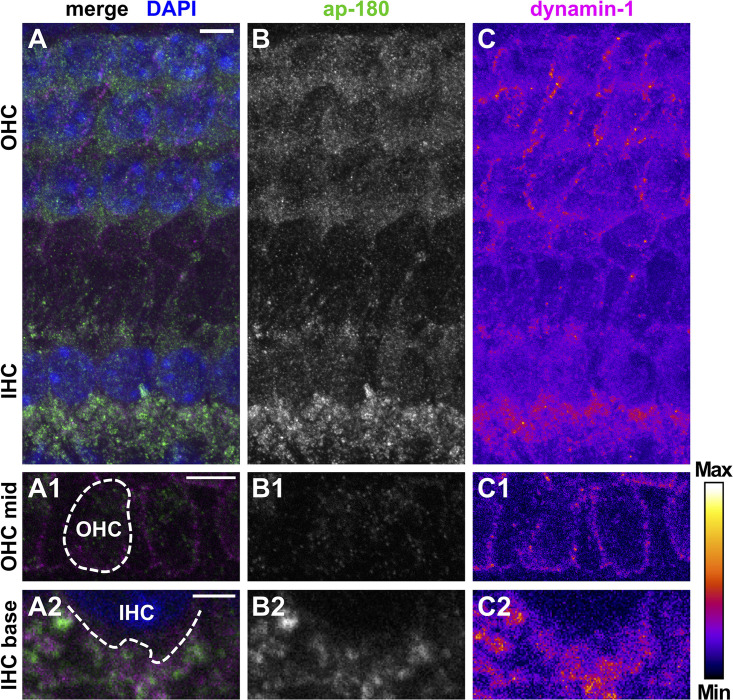
Protein expression of dynamin-1 and the endocytic protein ap-180 in neuronal and sensory cells of the pre-mature organ of Corti. Double immunolabeling of a typical pre-mature OC from a 4-day-old mouse shows predominant expression of dynamin-1 (**A**, magenta; **C**, intensity-coded) and ap-180 (**A**, green; **B**, gray scale) in NFs below IHCs and, to a much lesser extent, in OHCs and IHCs. Single optical slices of the OHC mid region **(A1–C1)** and the IHC base **(A2–C2)** are shown below at higher magnification: Dynamin-1 labeling was enriched at the lateral membrane of OHCs **(C1)**, whereas ap-180 labeling was weak and mainly present in their soma **(B1)**. The enlarged IHC base illustrates that the staining of the NFs beneath the weakly labeled IHCs was not overlapping between ap-180 and dynamin-1 **(A2–C2)**. Scale bars: 5 μm **(A–C; A1–C1)** and 2 μm **(A2–C2)**. Nuclei in the merged MIZP image **(A)** are labeled with DAPI.

**FIGURE 6:**
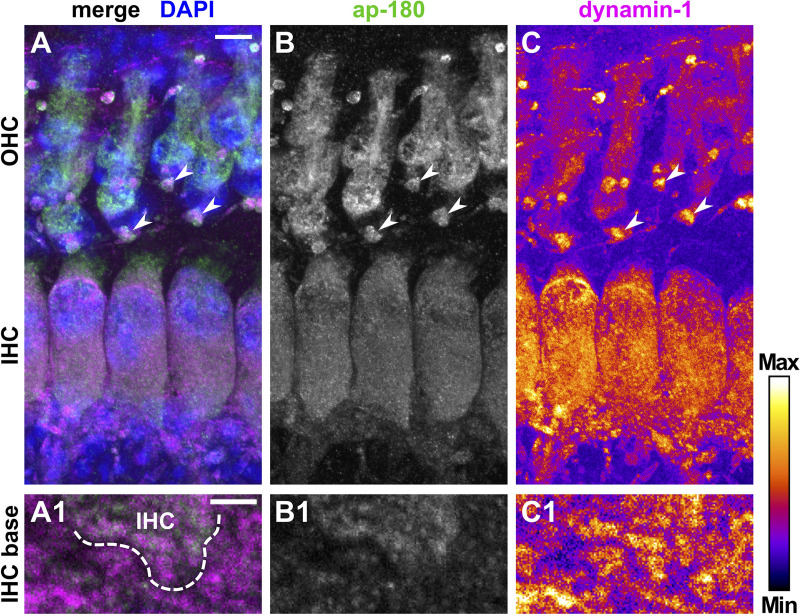
Increased expression of dynamin-1 and ap-180 in hair cells after the onset of hearing. Merged MIZP of a typical mature OC from a 20-day-old mouse immunolabeled for ap-180 (**A**, green; **B**, gray scale) and dynamin-1 (**A**, magenta; **C,** intensity-coded) and a single slice image of the IHC base at higher magnification **(A1–C1)**. Ap-180 staining was strong in the soma of IHCs and OHCs, but weaker in NFs **(B,B1)**. The intensity of dynamin-1 labeling was similar between IHCs and adjacent NFs, but slightly weaker in OHCs **(C,C1)**. Strong staining was found for both proteins in large structures below OHCs (arrowheads in **A–C**). Scale bars: 5 μm **(A–C)** and 2 μm **(A1–C1)**. Merged MIZP in **(A)** is shown with DAPI-labeled nuclei.

Dynamin-1 was co-labeled for adaptor protein (ap)-180, an endocytic protein in the mature cochlea that served here as a marker of HCs and SGN fibers/somata (Figures 5, 6; Kroll et al., 2020). Specificity of the binding of secondary antibodies was validated by omitting the dynamin-1 antibody (Supplementary Figure 4). In the pre-mature OC, dynamin-1 as well as ap-180 staining was sparse in IHCs (Figures 5A–C), compared to the distinct, non-overlapping labeling of both proteins in adjacent NFs (Figures 5A2–C2). In OHCs, dynamin-1 was enriched in the lateral but not the apical or basal membrane of the cell (Figures 5C,C1), while ap-180 was mainly present in the soma (Figures 5B,B1). Upon maturation, the expression pattern of both ap-180 and dynamin-1 underwent profound modifications (Figure 6). Ap-180 expression was now higher in IHCs and OHCs than in adjacent NF (Figures 6B,B1). In contrast, dynamin-1 was equally and highly expressed in IHCs and NF, but remained at low levels in OHCs, where it now resided in the soma rather than the lateral membrane (Figures 6C,C1). NFs below IHCs of both age groups likely represent afferent fibers of SGN, which also expressed both dynamin-1 and ap-180 (Supplementary Figure 5). Note that the strongly labeled round structures below OHCs are not part of the OHCs (Figures 6A–C, arrowheads), but most likely represent terminals of medial olivocochlear efferents (Spoendlin, 1985).

**FIGURE 7:**
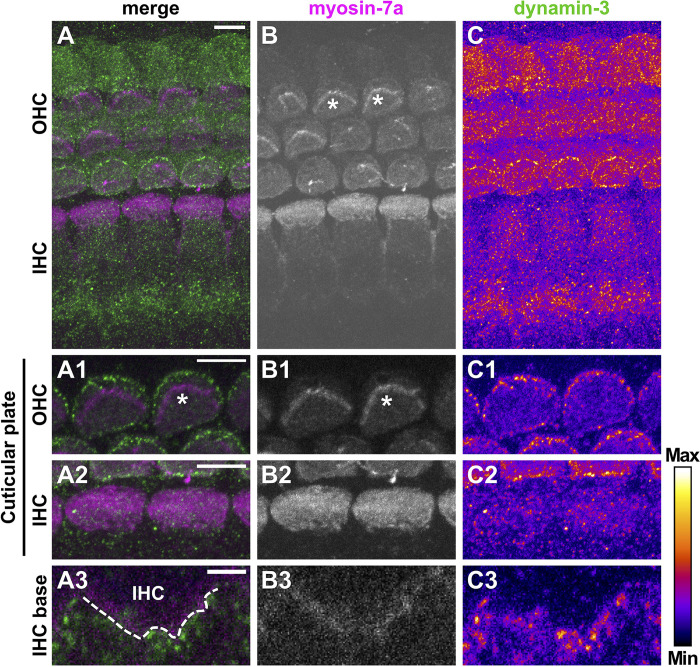
Dynamin-3 is highly expressed at cuticular plates of pre-mature OHCs. Double immunolabeling for the HC marker myosin-7a (**A**, magenta; **B**, gray scale) and dynamin-3 (**A**, green; **C**, intensity coded) in a typical pre-mature OC from a 4-day-old mouse. The overview MIZP image shows that dynamin-3 labeling is strongest in OHCs and beneath IHCs, but weak in IHCs **(A,C)**. A higher magnification MIZP of the cuticular plates and stereocilia (some of which indicated by asterisks) of two cells reveals distinct dot-like dynamin-3 labeling at the border of cuticular plates of OHCs **(C1)**, but not IHCs **(C2)**, which was clearly separate from the stereocilia expressing myosin-7a **(B1,B2)**. Single optical slices of the IHC base revealed that dynamin-3 was virtually absent from a myosin-7a-positive IHC, but present in the fibers beneath **(A3–C3)**. Scale bars: 5 μm **(A–C; A1/2–C1/2)** and 2 μm **(A3–C3)**.

**FIGURE 8:**
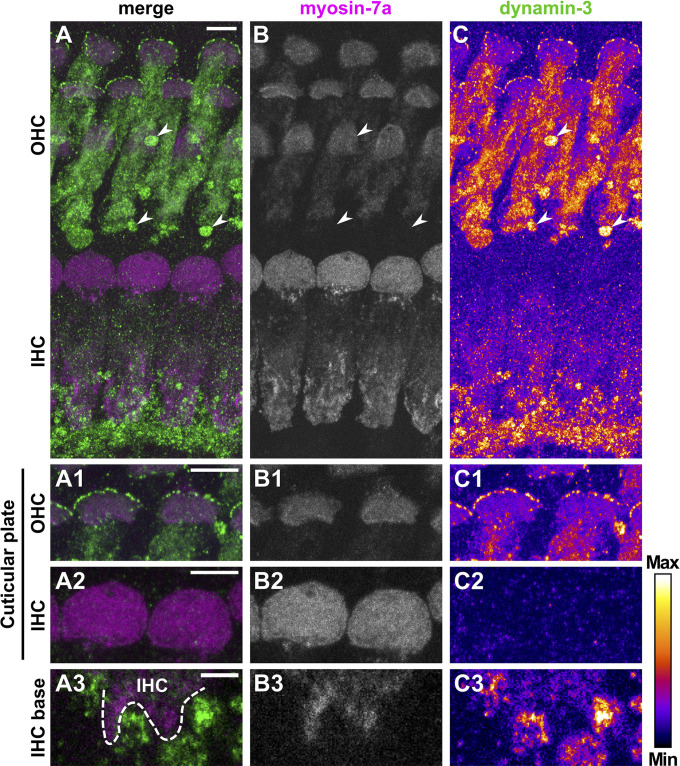
Dynamin-3 is mainly expressed in OHCs and nerve fibers of the mature organ of Corti. Double immunolabeling of a typical mature OC from a 20-day-old mouse for myosin-7a (**A**, magenta; **B**, gray scale) and dynamin-3 (**A**, green; **C**, intensity-coded) shows predominant labeling of dynamin-3 in OHCs and fibers beneath IHCs, while IHCs are barely stained **(A,C)**. Strong dynamin-3 staining was present below OHCs in a structure devoid of myosin-7a (arrowheads in **A**–**C**). A detailed MIZP of cuticular plates and stereocilia reveals dot-like dynamin 3 signals at the strial border of the myosin-7a-positive cuticular plates of OHCs **(A1–C1)**, but not IHCs **(A2–C2)**. A single optical slice of the IHC base illustrates that dynamin-3 expression was strong in NFs but weak in adjacent myosin-7a-positive IHCs **(A3–C3)**. Scale bars: 5 μm **(A–C; A1/2–C1/2)** and 2 μm **(A3–C3)**.

Dynamin-3 expression was assessed together with the HC marker myosin-7a (Li et al., 2020). Again, negative controls confirmed specific binding of secondary antibodies to the dynamin-3 antibody (Supplementary Figure 4). In the OC of pre-hearing mice (Figure 7), dynamin-3 was predominantly found in OHCs (Figures 7C,C1) and NF below IHCs (Figure 7C3) but was absent from IHCs (Figures 7C2,C3). In pre-mature OHCs, a ring of distinct dynamin-3 dots circumscribed the entire cuticular plates forming the apical border of HCs (Figure 7C1), which was clearly separated from the myosin-7a labeled stereocilia (asterisks in Figures 7A1,B1). While the general distribution of dynamin-3 and myosin-7a was maintained upon maturation (Figure 8), the intensity of dynamin-3 labeling was increased in OHCs (Figure 8C) and NF below IHCs (Figure 8C3) compared to the low signal in IHCs (Figures 8C,C2,C3). The dynamin-3 labeled NFs most likely represent afferent fibers originating in SGN somata, which showed dynamin-3 but were devoid of the HC marker myosin-7a (Supplementary Figures 5B2,B3). Similar to dynamin-1, dynamin-3 was expressed in the myosin-7a-negative efferent terminals below OHCs (Figures 8A–C, arrowheads).

In conclusion, we found that dynamin-1 and ap-180, which were highly expressed in mature IHCs, were largely absent in pre-mature IHCs of early postnatal mice. In contrast, dynamin-3 was mainly found in the soma of OHCs of both age groups, where it was further enriched at the border of cuticular plates. Neuronal expression of all three endocytic proteins was evident from NF and SGN labeling.

## Discussion

### Whole-Mount FISH in the Pre-mature and Mature Organs of Corti

In the present study, we established a workflow of RNAscope^®^ *FISH* that enabled us to subcellularly localize and quantify mRNA transcripts of three genes at once in single HCs without removing them from the tissue. Furthermore, the whole-mount approach has the advantage over sections that mRNA transcript numbers of the entire cell volume instead of a single plane can be determined and compared between neighboring cells, e.g., between neighboring IHCs, or between IHCs and OHCs.

To our knowledge, this is the first study providing comparative quantification of mRNA transcripts on single-cell level in the pre-mature and mature whole-mount OC. Reliable quantification required careful adjustment of our experimental conditions for each age group to a point, where the number of fluorescent signals was as high as possible in the positive control signal and, simultaneously, as low as possible in the negative control. We obtained adequate high amounts of mRNA transcripts of the housekeeping genes *polr2a*, *ppib*, and *ubc* in both pre-mature and mature OCs. The age-dependent expression changes of the housekeeping genes might be due to a different metabolism between the highly dynamic pre-mature OC, which undergoes major rebuilding and the rather rigid mature OC that soon after the onset of hearing does no longer undergo major changes. However, no studies were available comparing these housekeeping genes in the OC as a function of age.

Despite a generally low signal in our negative controls, differences between the individual detection channels were found for pre-mature IHCs; we thus did not compare between target probes. Since the probes for all three detection channels of our negative controls were specific for the same mRNA, *dapb*, the different signal numbers between these channels are likely to be related to the associated fluorophores. In preliminary experiments, when using alternative fluorophore combinations with the three detection channels, we in fact found that signal numbers generally were higher in the detection channel that was combined with Alexa 647 (not shown). Considering possible low mRNA transcript numbers of target genes, such as low *dnm1* mRNA content of adult OHCs found in the present study, non-specific signals might cause overestimation of the actual transcript numbers, the risk of which we reduced by normalization to negative controls. These results emphasize the importance of negative controls in every experiment.

### mRNA Transcription Profiles of Endocytic Proteins in the OC

At both age groups investigated, *dnm1* and *sh3gl2* mRNA was enriched in IHCs, whereas *dnm3* was mainly present in OHCs. This confirms previous studies analyzing the transcriptome of OCs using RNA-sequencing or DNA-microarrays (Liu et al., 2014; Cai et al., 2015; Li et al., 2018; Wiwatpanit et al., 2018). Both techniques involved pooling of isolated cells in groups of ≥ 700 cells and could therefore not provide information about the transcription profiles of single cells and their exact location within the tissue.

The differences between the transcription profiles of pre-mature IHCs and OHCs further increased upon maturation. After terminal mitosis at embryonic day 14–15 in mice, newly formed IHCs and OHCs are physiologically very similar, but start to differentiate soon after birth in order to adapt their physiology and morphology to the respective functions in the mature OC (Housley et al., 2006). This process is partly driven by general master switches expressed in both HC types (Kuhn et al., 2011), but also by transcription factors exclusive to one type of HC (Wiwatpanit et al., 2018). The age-dependent diversification of *dnm1* mRNA transcription rates between IHCs and OHCs was not only evident by an increase in IHCs, but by a concomitant reduction in OHCs. This process might be driven by regulators, such as the zinc finger protein insm1, which is transiently expressed in nascent OHCs, where it suppresses upregulation of certain genes enriched in mature IHCs (Wiwatpanit et al., 2018).

### Protein Expression Corroborates mRNA Transcription Profiles

Using immunohistochemistry, we analyzed if the spatiotemporal transcription profile of *dnm1* and *dnm3* was reflected in dynamin-1 and -3 protein expression and where the protein was localized in the cell.

Dynamin-1 expression was equally high in mature IHCs and adjacent NFs confirming previous studies (Neef et al., 2014), whereas it was low in OHCs of the same age, thereby reflecting the difference in transcription rates of *dnm1* in HCs. In contrast to dynamin-1 protein, *dnm1* mRNA was absent from NFs at IHCs, whereas it was enriched in the somata of SGNs (Supplementary Figure 3). This suggests that dynamin-1 protein is translated from *dnm1* mRNA in the SGN soma and then transported along the axon to its destination at the afferent terminals innervating IHCs.

Despite high *dnm1* mRNA content in pre-mature IHCs, expression of dynamin-1 protein was low at this age, when compared to the strong dynamin-1 signal in mature IHCs. Accordingly, high levels of *dnm1* mRNA but low expression of dynamin-1 protein have been described in embryonic skeletal muscle, whereas dynamin-1 protein levels were high after maturation (Noakes et al., 1999). Similarly, dynamin-1 protein abundance rises substantially during the maturation of neuronal synapses (Fan et al., 2016). In nerve terminals, efficiency of endocytosis rises with age and maturity level, which is accompanied by an increasing abundance of endocytic proteins, that might prevent exhaustion of the endocytic apparatus in mature nerve terminals even after intense activity (Renden and von Gersdorff, 2007; reviewed in Milosevic, 2018). This indicates that during the highly dynamic period of terminal differentiation of the OC, high *dnm1* mRNA levels precede the increase in dynamin-1 protein abundance to the high levels of mature IHCs. A detailed analysis of the time course of *dnm1* mRNA and dynamin-1 protein expression might help to support this hypothesis.

In contrast to dynamin-1, dynamin-3 was predominantly expressed in both pre-mature and mature OHCs confirming enrichment of *dnm3* mRNA in this cell type. While *dnm3* mRNA content was reduced with age, dynamin-3 expression appeared stronger in the soma of mature OHCs. This might indicate that high *dnm3* transcription and dynamin-3 translation rates at P4 precede the visible accumulation of dynamin-3 protein. Moreover, protein turnover of dynamin-3 might be slow in mature OHCs, which would be reflected in low *dnm3* mRNA levels.

Li et al. (2018) reported predominant expression of dynamin-3 in OHCs, which was, however, restricted to their stereocilia, thereby contrasting the labeling of the border of cuticular plates and the soma of OHCs in the present study. This might be explained by the use of another dynamin-3 antibody, which might have different sensitivities to the several splice variants of *dnm3* (Cao et al., 1998) that might be expressed in OHCs.

Neef et al. (2014) found expression of dynamin-3 in IHCs. Using the same dynamin-3 antibody, we found that, independent from mouse age and fixative (Zamboni or ethanol), IHCs showed weak labeling for dynamin-3 in seven out of 32 experiments, when the primary antibody was incubated overnight. IHCs were devoid of dynamin-3 labeling (10 out of 10 experiments) following brief incubation. This might reflect low dynamin-3 expression in IHCs requiring extended incubation of the antibody, which is supported by a low *dnm3* mRNA content in IHCs in the present study and lack of *dnm3* mRNA in IHCs of adult Kunming mice (Cai et al., 2015).

From our experiments, we cannot differentiate, if dynamin-3 expression in NFs is localized in afferent or efferent fibers. However, the *dnm3* mRNA content in SGNs was low compared to that of *dnm1* and *sh3gl2* suggesting that *dnm3* might be preferentially expressed in efferent terminals.

*Sh3gl2* transcription profiles could not be complemented by immunohistochemical analysis of endophilin-A1 expression since no antibody is so far functional in the cochlea (not shown; Kroll et al., 2019). While endophilin-A1 expression was found by immunoblots of mature cochlear lysates (Kroll et al., 2019), information of the cellular distribution is therefore still pending.

### Possible Function of Dynamin-1, Dynamin-3, and Endophilin-A1 in the OC?

Our data show that dynamin-1 is mainly expressed in mature IHCs, but low in pre-mature IHCs at postnatal day 4. While the onset of synaptic transmission of IHCs is before birth, release rates are still low at P4 compared to the mature cell (Beutner and Moser, 2001; Johnson et al., 2005). This suggests that at this age slow rates of endocytosis and thus low amounts of endocytic proteins are required in pre-mature IHCs (Renden and von Gersdorff, 2007).

Spatiotemporal regulation of *sh3gl2* transcription was here similar to that of *dnm1*, which supports a coupled function and regulation of dynamin-1 and endophilin-A1. A functional role of dynamin-1 and endophilin-A1 in endocytosis of vesicle membrane of mature IHCs has been demonstrated before (Duncker et al., 2013; Neef et al., 2014; Kroll et al., 2019). Dynamin mediates fission of the internalized membrane and is recruited by its binding partner endophilin to the endocytic site, supporting an interconnected function and possible common regulation during terminal differentiation of IHCs (Sundborger and Hinshaw, 2014).

The function of dynamin-1 and endophilin-A1 in OHCs has never been investigated. A point mutation in the *Dnm1* gene of *fitful* mice resulting in reduced dynamin-1 function did not affect distortion product otoacoustic emissions indicating intact OHC amplifier function despite elevated hearing thresholds (Boumil et al., 2010), indicating that expression of dynamin-1 at the lateral membrane of pre-mature OHCs is non-essential for the amplifier function of mature OHCs. Alternatively, dynamin-1 and endophilin-A1 might both be involved in refinement of the OHC afferent pathway driven by spontaneous activity and thus synaptic release of early postnatal OHCs (Ceriani et al., 2019). After the onset of hearing, only few unmyelinated type II afferent fibers innervate OHCs, which are activated upon noise trauma and have thus been suggested to serve as cochlear nociceptors reporting cochlear damage (Flores et al., 2015; Liu et al., 2015). This infrequent activation of OHC synapses requires less efficient mechanisms of vesicle reformation and hence less dynamin-1 and endophilin-A1 as reflected in our data.

Here, dot-like expression of dynamin-3 was present near the edge of cuticular plates of pre-mature and mature OHCs. Activity-independent endocytosis has been recorded at the apex of guinea-pig OHCs (Griesinger et al., 2004; Harasztosi and Gummer, 2019). As shown by electron microscopy on bullfrog vestibular HCs, vesicles are most likely retrieved at the border of bullfrog vestibular HCs between cuticular plates and tight junctions (Kachar et al., 1997). Similarly, cytoplasmic vesicles have been found at the apical border of lateral line sensory HCs of the zebrafish (Seiler and Nicolson, 1999). In pre-mature as well as mature OHCs, dynamin-3 might be involved in this activity-independent form of endocytosis. The lack of expression at the cuticular plate of IHCs offers the possibility of a previously unknown unique function of OHCs in the cochlea. Its high expression at the cuticular plate of OHCs and absence from IHCs indicates that dynamin-3 might not be involved in activity-dependent endocytosis of HCs. However, since dynamin-3 is furthermore expressed in the soma of OHCs, we cannot completely exclude its involvement in synaptic vesicle reformation.

## Data Availability Statement

The raw data supporting the conclusions of this article will be made available by the authors, without undue reservation.

## Ethics Statement

The animal study was reviewed and approved by Landratsamt für Verbraucherschutz Amtstierärztlicher Dienst, Saarbrücken, Germany.

## Author Contributions

SE conceived and supervised the study, wrote the manuscript, and was responsible for funding acquisition and project administration. GH acquired the data and reviewed and edited the manuscript. Both authors analyzed and visualized the data.

## Conflict of Interest

The authors declare that the research was conducted in the absence of any commercial or financial relationships that could be construed as a potential conflict of interest.

## Abbreviations

FISH: fluorescent *in situ* hybridization
HC: hair cell
IHC: inner hair cell
MIZ(X)P: maximum intensity *z*-axis (*x*-axis) projection
NF: nerve fiber
OC: organ of Corti
OHC: outer hair cell
SGN: spiral ganglion neuron.
