# Supplementary material for: Quantitative Fluorescent in situ Hybridization Reveals Differential Transcription Profile Sharpening of Endocytic Proteins in Cochlear Hair Cells Upon Maturation
Source: Front Cell Neurosci. 2021 Feb 26;15:643517. doi: 10.3389/fncel.2021.643517 (PMC7952526; doi:10.3389/fncel.2021.643517)
Supplement: Supplementary file 1 [file Data_Sheet_1.PDF]

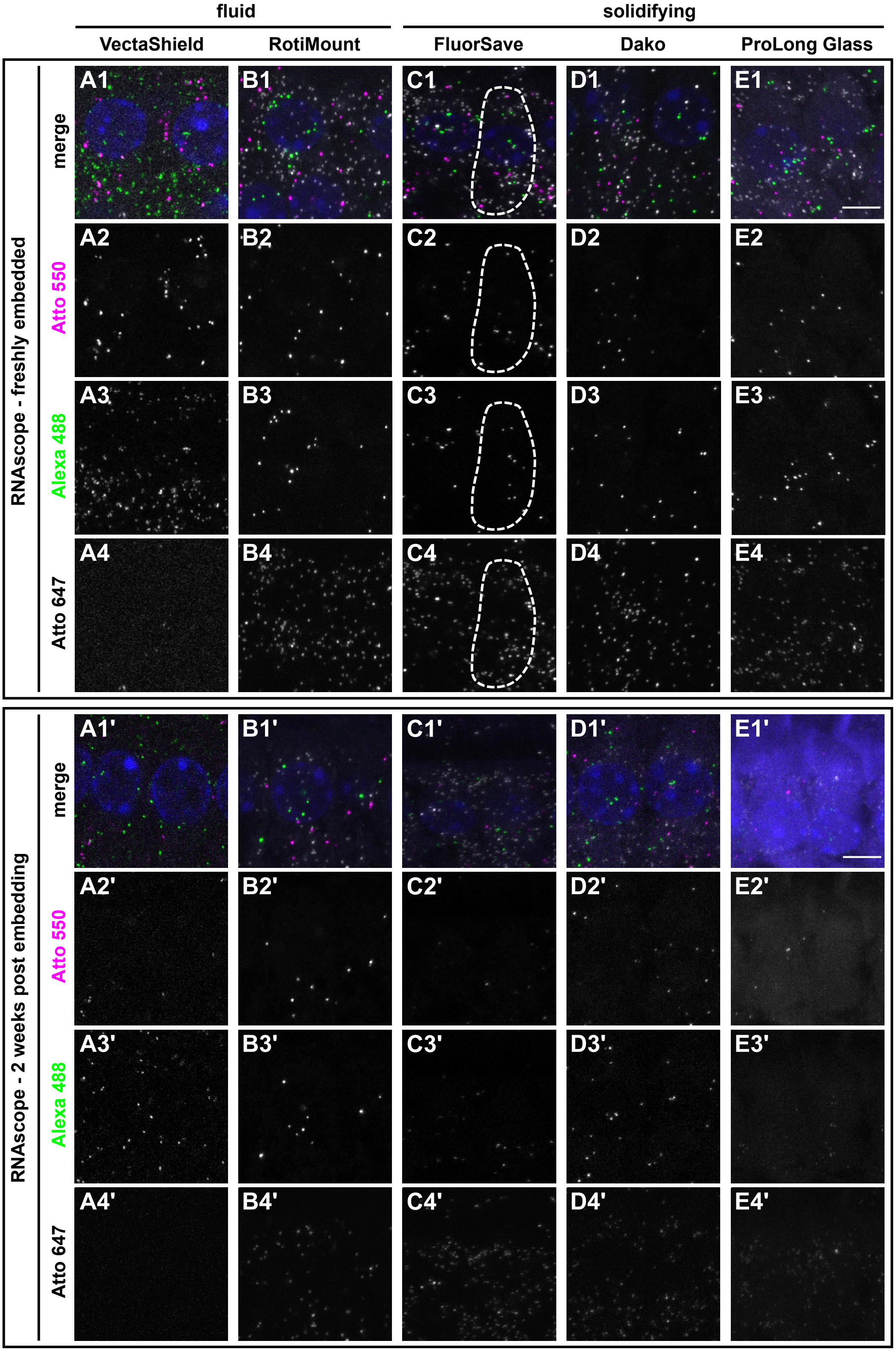

Supplementary Figure 1. Effect of different embedding media on RNAscope® quality. **Upper panel:** Whole-mount RNAscope® images taken shortly after (0-3 days) embedding with fluid phase embedding media VectaShield (**A**) or RotiMount (**B**) or with solidifying embedding media FluorSave (**C**), Dako (**D**) or ProLong Glass Antifade (**E**). Projections of confocal images of two IHCs are shown, the shape of a representative IHC is indicated by a dotted line in **C**. Positive control probes detected mRNA of *ppib* in the Atto-550 (**B2**-**E2**), *polr2a* in the Alexa-488 (**B3**-**E3**), and *ubc* in the Atto-647 channel (**B4**-**E4**), except for VectaShield (Atto-550: *polr2a*, **A2**; Alexa-488: *ubc*, **A3**; Alexa-647, *ppib*, **A4**). Nuclei in merged images are labeled

with DAPI (**A1-E1**). Tissue morphology was well preserved with all embedding media. Note that Atto-647 fluorescence was quenched by VectaShield (**A4**) and reduced by ProLong Glass (**E4**) when compared to the other embedding media (**B4-D4**). **Lower panel:** Specimens were re-analyzed 2-3 weeks later for preservation of fluorescence by the different embedding media. Images were taken using identical settings as before. Atto-550 fluorescence was reduced in all embedding media (**A2'**, **C2'-E2'**) except RotiMount (**B2'**). Alexa-488 fluorescence was reduced in FluorSave (**C3'**) and to a great extend in ProLong Glass (**E3'**), but mostly preserved in the other media (**A3'**, **B3'**, **D3'**). Atto-647 fluorescence was absent in VectaShield (**A4'**) and reduced in the remaining embedding media (**B4'-E4'**), where, however, fluorescence could still be analyzed by increasing the gain settings of the microscope (not shown). Note the high background labeling for DAPI and, to a lower degree, Atto-550 and Alexa-488 in ProLong Glass (**E1'-E3'**). Scale bars: 5 µm.
