# Supplementary material for: Quantitative Fluorescent in situ Hybridization Reveals Differential Transcription Profile Sharpening of Endocytic Proteins in Cochlear Hair Cells Upon Maturation
Source: Front Cell Neurosci. 2021 Feb 26;15:643517. doi: 10.3389/fncel.2021.643517 (PMC7952526; doi:10.3389/fncel.2021.643517)
Supplement: Supplementary file 2 [file Data_Sheet_2.PDF]

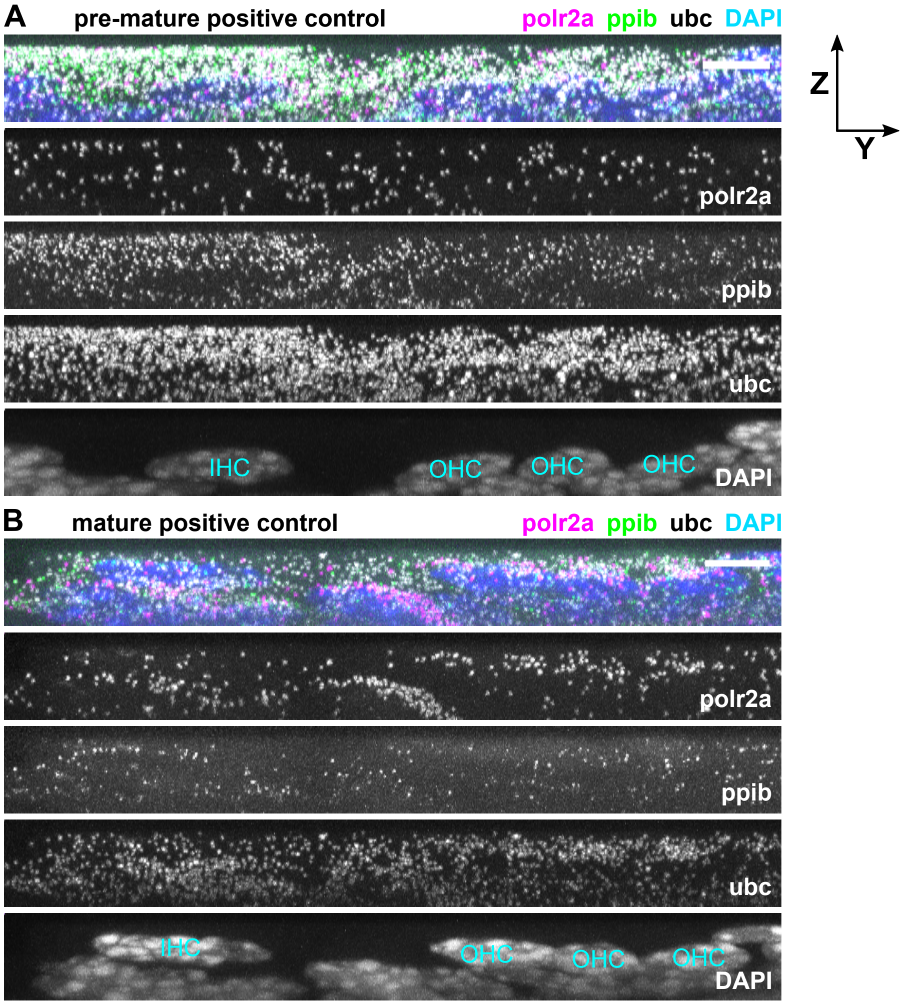

Supplementary Figure 2. Distribution of RNAscope® positive control signals in the OC. X-Axis projection of an IHC and neighboring three OHCs of a pre-mature (**A**) and a mature OC (**B**). RNAscope® signals of the positive control probes indicated in the graph are shown. For clarity, DAPI-stained nuclei labeled by cell type are shown at the respective bottom graph. Note that in both age groups mRNA dots are not specifically enriched in hair cells. Extension of Z-axis is inflated and not true to the scale bar. Diagram on the right indicates axes of the graphs. Scale bar: 5 µm.
