# Supplementary material for: Quantitative Fluorescent in situ Hybridization Reveals Differential Transcription Profile Sharpening of Endocytic Proteins in Cochlear Hair Cells Upon Maturation
Source: Front Cell Neurosci. 2021 Feb 26;15:643517. doi: 10.3389/fncel.2021.643517 (PMC7952526; doi:10.3389/fncel.2021.643517)
Supplement: Supplementary file 3 [file Data_Sheet_3.PDF]

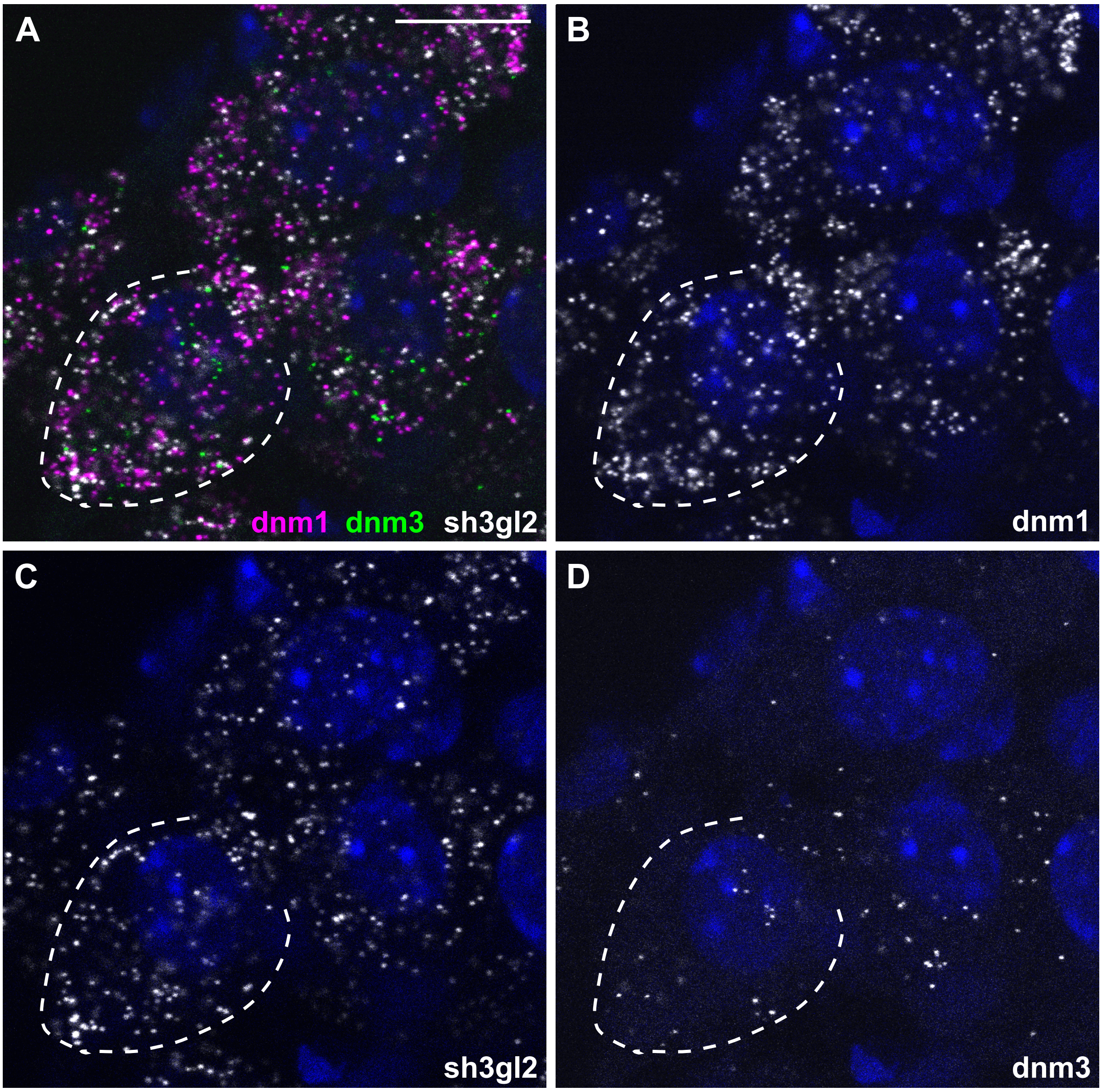

Supplementary Figure 3. Expression of endocytic mRNA in SGN. Single plane image shows non-overlapping expression (**A**, merge) of target mRNAs from *dnm1* (**B**), *sh3gl2* (**C**) and *dnm3* (**D**) in SGN somata. Note the very low *dnm3* content as compared to *dnm1* and *sh3gl2*. The cell body of a single SGN is indicated by the dotted line. Nuclei are stained with DAPI. Scale bar 10 µm.
