# Supplementary material for: Quantitative Fluorescent in situ Hybridization Reveals Differential Transcription Profile Sharpening of Endocytic Proteins in Cochlear Hair Cells Upon Maturation
Source: Front Cell Neurosci. 2021 Feb 26;15:643517. doi: 10.3389/fncel.2021.643517 (PMC7952526; doi:10.3389/fncel.2021.643517)
Supplement: Supplementary file 4 [file Data_Sheet_4.PDF]

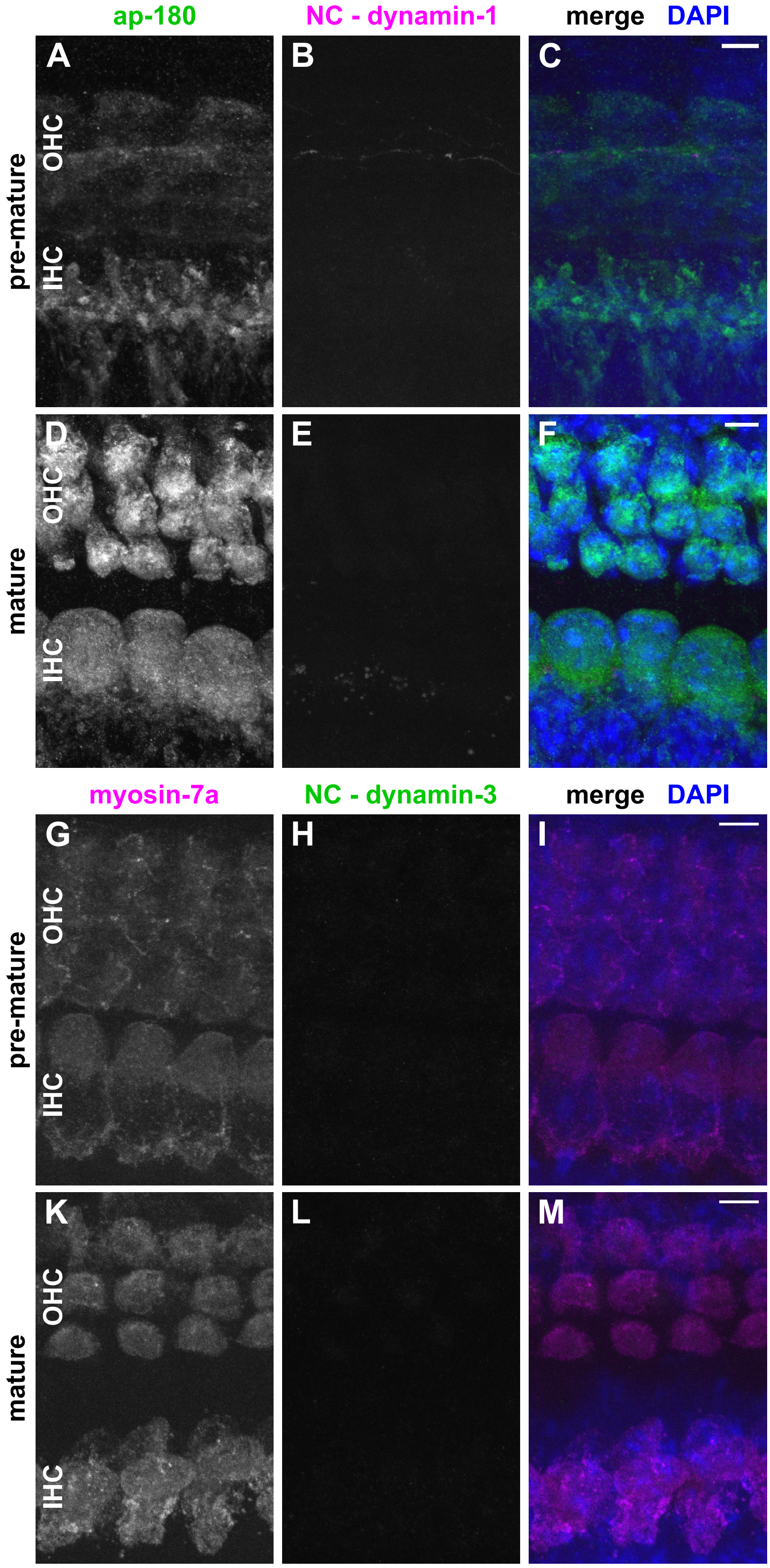

Supplementary Figure 4. Negative controls of immunolabeling shown in figure 5-8 (main text). Tissue was not incubated with dynamin-1 (pre-mature **B**, mature **E**) and dynamin-3 antibody (pre-mature **H**, mature **L**), respectively. Lack of labeling revealed specific binding of secondary antibodies to the dynamin-1 and dynamin-3 antibody in pre-mature and mature OCs. Hair cells were counterstained with ap-180 (**A, D**) or myosin-7a (**G, K**).
