# Supplementary material for: Quantitative Fluorescent in situ Hybridization Reveals Differential Transcription Profile Sharpening of Endocytic Proteins in Cochlear Hair Cells Upon Maturation
Source: Front Cell Neurosci. 2021 Feb 26;15:643517. doi: 10.3389/fncel.2021.643517 (PMC7952526; doi:10.3389/fncel.2021.643517)
Supplement: Supplementary file 5 [file Data_Sheet_5.PDF]

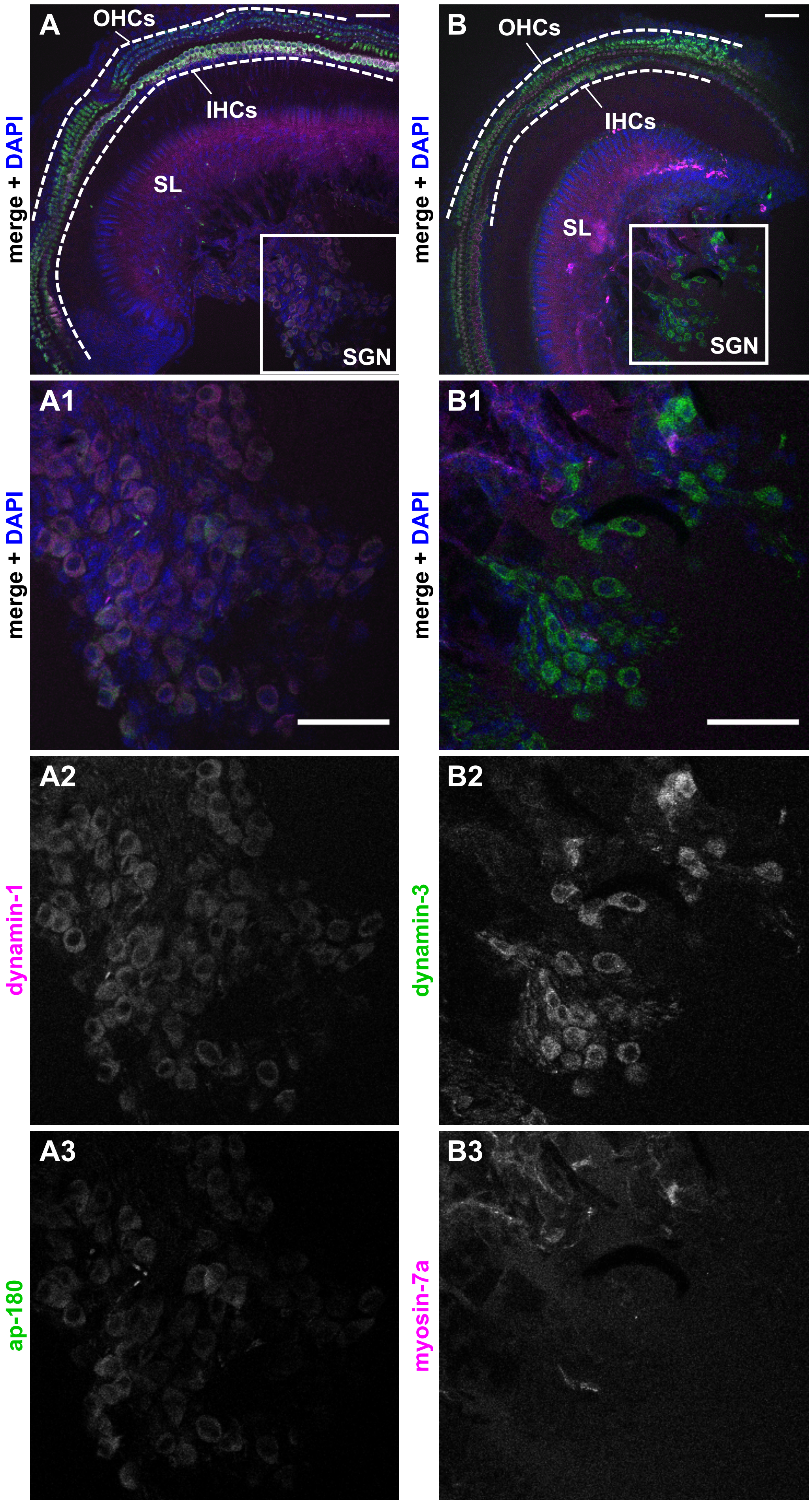
A
OHCs
IHCs
SL
SGN
merge + DAPI
B
OHCs
IHCs
SL
SGN
merge + DAPI
A1
merge + DAPI
B1
merge + DAPI
A2
dynamin-1
B2
dynamin-3
A3
ap-180
B3
myosin-7a

Supplementary Figure 5. Protein expression of dynamin-1 and -3 in SGN**.** MIZPs of mature OCs labeled for dynamin-1 (**A**) and -3 (**B**). (**A**, **B**) Overview of apical-turn OCs with one row of inner (IHCs) and three adjacent rows of outer hair cells (OHCs) and spiral ganglion neurons (SGN). Note that the staining of the spiral limbus (SL) was also present in negative controls (not shown) and is thus considered non-specific. Enhanced images show co-labeling (**A1**) of SGNs for dynamin-1 (**A2**) and ap-180 (**A3**). dynamin-3 (**B1, B2**) but not myosin-7a (**B3**) is also expressed in SGN. Scale bars 50 μm.
